# Supplementary material for: Computational Modelling and Sustainable Synthesis of a Highly Selective Electrochemical MIP-Based Sensor for Citalopram Detection
Source: Molecules. 2022 May 21;27(10):3315. doi: 10.3390/molecules27103315 (PMC9143463; doi:10.3390/molecules27103315)
Supplement: Supplementary file 1 [file molecules-27-03315-s001.zip › molecules-1694769-supplementary.pdf]

molecules

MDPI

Supplementary Materials

Computational modelling and sustainable synthesis of a highly selective electrochemical MIP-based sensor for citalopram detection

Patrícia Rebelo [1,2], João G. Pacheco [1,*], Iuliia V. Voroshylova [2,*], Isabel Seguro [1], M. Natália D.S. Cordeiro [2], Cristina Delerue-Matos [1]

[1] REQUIMTE, LAQV, Instituto Superior de Engenharia do Porto, Instituto Politécnico do Porto, Rua Dr. António Bernardino de Almeida 431, 4200-072 Porto, Portugal

[2] REQUIMTE, LAQV, Departamento de Química e Bioquímica, Faculdade de Ciências, Universidade do Porto, Rua do Campo Alegre, s/n, 4619-007 Porto, Portugal

* Correspondence: jpgpa@isep.ipp.pt (J.G.P); voroshylova.iuliia@fc.up.pt (I.V.V)

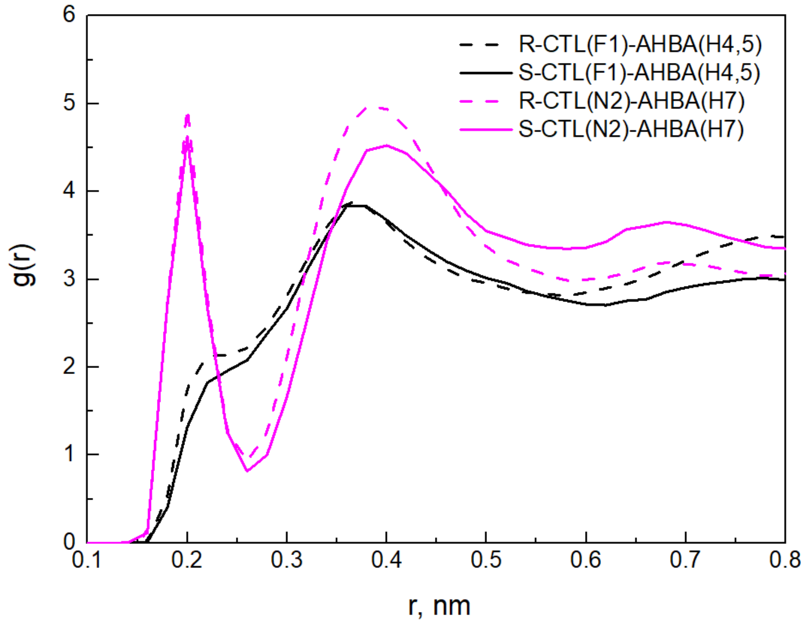

Figure S1. Radial distribution functions, g(r), between selected sites of R(-)CTL, S(+)CTL and AHBA molecules.

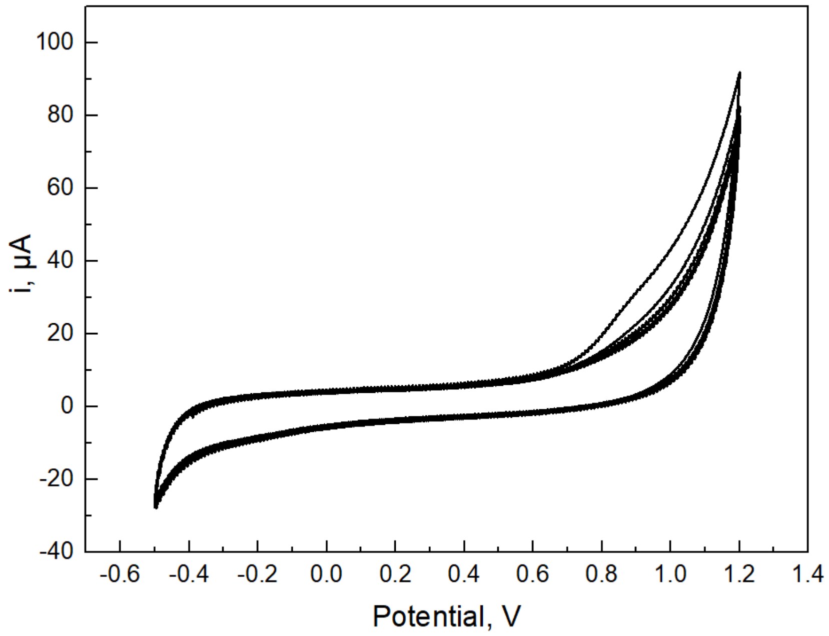

Figure S2. Cyclic voltammograms of the MIP SPCE extraction in phosphate buffer solution (0.1 M, pH 7).
